# A study on preparation of modified Graphene Oxide and flame retardancy of polystyrene composite microspheres

**DOI:** 10.1080/15685551.2020.1720934

**Published:** 2020-01-28

**Authors:** Yazhen Wang, Yingbo Qing, Yu Sun, Meng Zhu, Shaobo Dong

**Affiliations:** a College of Chemical and Chemical Engineering, Qiqihar University, Qiqihar, China; b College of Chemistry, Chemical Engineering and Resource Utilization, Northeast Forestry University, Harbin, Heilongjiang, China; c Heilongjiang Province Key Laboratory of Polymeric Composition Material, Qiqihar, China; d College of Materials Science and Engineering, Qiqihar University, Qiqihar, China

**Keywords:** DOPO, Graphene Oxide, suspension polymerization, polystyrene, flame retardant

## Abstract

In this paper, the ODOPM, a kind of 9, 10-dihydro-9-oxygen-heterooxy-10-phosphoro-10-oxygen (DOPO) derivative, was obtained by hydroxylation of DOPO. Further, a phosphorus nano-flame retardant (GO-ODOPM) was obtained by addition reaction with carboxylated Graphite Oxide (GO-COOH). And then Graphene Oxide/polystyrene (GO-ODOPM/PS) composite microspheres were obtained via suspension polymerization of styrene with GO-ODOPM. The decrease of the peak heat release rate (HRR) and total heat release rate (THR) for the GO-ODOPM/PS composite microspheres was obtained when the content of the additives was only 3.0 wt% is more than 36.2% and 33.6% compared with the pure PS microspheres, respectively. Thermogravimetric (TG), dynamic rheology and carbon residue analysis were used to study the flame-retardant mechanism of GO-ODOPM in PS microspheres. The results revealed that the addition of GO-ODOPM obviously reduced the fire hazard of polystyrene (PS) microspheres. Thus, this work provided a feasible method to design efficient flame retardants for enhancing fire safety of polymers.

1.

## Introduction

As one of the five most commonly used engineering thermoplastics, polystyrene (PS) is widely used in the electrical, decorative, construction and transportation industries, and military industries, due to its lightweight, low cost, good corrosion resistance, good insulation, high transparency and easy processing [1]. However, PS is extremely flammable, and a large amount of heat was released during the combustion process accompanied by a large amount of smoke, which greatly threatens people’s lives and property. Therefore, improving the thermal stability and flame retardancy of PS has become an important research direction of current researchers [2–3].

At present, flame-retardant modification of polystyrene and other polymer materials is mainly realized by adding flame retardant. The oxygen index, thermal stability, heat release rate and smoke density of the polymer materials were significantly improved after adding flame retardant. However, the traditional halogen flame-retardant decomposition in the combustion process produced a lot of smoke and corrosive toxic halogen gas (HCL, HBr, etc.), which will cause secondary damage. Therefore, it is particularly important to develop a low toxicity and good performance halogen-free flame retardant. DOPO is an important halogen-free flame retardant with low toxicity, good heat resistance and flame-retardant properties, due to its biphenyl ring and phenanthrene structure. During the combustion process, DOPO is decomposed to form PO·, PO_2_· and other reactive radicals by heating, which can capture free radicals and O· generated by polymer and oxygen, so as to achieve the purpose of preventing combustion. In addition, DOPO could catalyze the formation of polymer coke and act as a synergistic flame retardant between gas phase and solidification phase [4]. Kim W research group [5] synthesized a series of DOPO derivatives FR and applied them to acrylonitrile-butadiene-styrene copolymer (ABS). The results showed that when the load of FR is 27.5–30.0 wt%, the flame-retardant performance of ABS/FR mixture can be significantly improved, which can reach V-0 rating, and smoke emission can be greatly reduced. However, when the amount of the flame retardant added is particularly large, it will lead to poor compatibility between flame retardant and polymer, affecting the mechanical properties of the polymer and the flame-retardant effect of the flame retardant. Therefore, the key problem in our current research is how to combine DOPO with other flame retardants to further improve the flame-retardancy efficiency [6].

Graphene is a monolayer of carbon atoms with a six-sided honeycomb network structure formed by the combination of one carbon atom and three adjacent carbon atoms. Due to its special structure, graphene has such excellent properties as high heat conduction, high conductivity and high specific surface area. According to research, when a small amount of graphene or its derivative is uniformly dispersed in the polymer matrix, it can effectively improve the heat resistance, mechanical and flame-retardant properties of the polymer [7,8]. Therefore, graphene and its derivatives are often used as a new halogen-free nano-flame retardant in polymers. Huang et al. [9] added Graphene Oxide (GO) into polyvinyl alcohol as a flame retardant to obtain flame-retardant composites. The test results showed that when the GO addition was 3 wt%, the maximum heat release rate decreased from 373 kW/m^2^ to 190 kW/m^2^, and the ignition time was greatly extended. However, due to the strong π–π interaction between the graphene layers, the graphene sheets are easily stacked and cannot fully exert their excellent properties. Therefore, it is necessary to organically modify the graphene. GO, as an important derivative of graphene, is beneficial to its organic modification because its surface contains a large number of reactive groups such as hydroxyl, carboxyl and epoxy groups [10].

With the development of high functional flame-retardant materials, single flame-retardant materials are not enough to meet their needs due to their own shortcomings. In order to improve the fire resistance of materials, the cooperative flame-retardant system has become a hot research direction of polymer flame retardant [9]. Therefore, the co-flame-retardant system of GO and ODOPM was adopted in this paper, and GO-ODOPM was applied to polystyrene microspheres through suspension polymerization to further study the thermal stability and flame-retardant properties of GO-ODOPM/PS composite microspheres.

## 2. Experimental

### 2.1. Materials

Absolute ethanol and formaldehyde solution (mass fraction 37%) were purchased from Tianjin Tianli Chemical Reagent Co., Ltd. (Tianjin, China). GO and deionized water were prepared in the laboratory. Styrene, analytically pure (>99%) was purchased from Beijing Chemical Plant (Beijing, China). Bromoacetic acid (C_2_H_3_BrO_2_), 1-ethyl-(3-dimethylaminopropyl) carbodiimide hydrochloride (EDC· HCL), 4-dimethylaminopyridine (DMAP) were obtained from Aladdin Biochemical Technology Co., Ltd (Shanghai, China). DOPO (purity 98%) was obtained from Alpha Fine Chemical Co., Ltd. (Jiaxing, China). N, N’-dimethylformamide (DMF), dibenzoyl peroxide (BPO) and sodium sulfite (Na_2_SO_3_) were purchased from Tianjin Kemiou Chemical Reagent Co., Ltd. (Tianjin, China). Polyvinyl alcohol (PVA-124) and polyvinyl alcohol (PVA-1788) were purchased from Beijing Jingzewang Chemical Co., Ltd. (Beijing, China).

### 2.2. Experimental sample preparation

#### 2.2.1. Synthesis of ODOPM

DOPO (0.5 mol, 108.0 g) and 250 mL of absolute ethanol were added into a 500 mL three-neck flask equipped with a reflux condenser and mechanical stirrer. The mixture was stirred and heated to 70°C until DOPO dissolved completely under a nitrogen atmosphere. Then, formaldehyde solution (37%, 45.0 g) was added into the reaction in 30 min. The resulting mixture was continuously stirred for 6 h at 85°C. After the reaction was completed, the solid product was filtered and washed with ethanol. The obtained ODOPM was then vacuum-dried at 80°C for 12 h. Seventy-five grams of white powder was obtained with a yield of 70% [11]. The synthetic route was shown in.

**Scheme 1.**
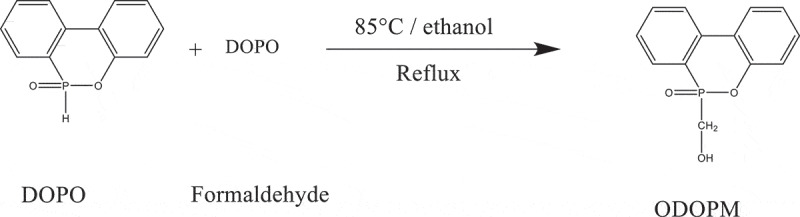
The synthesis process of ODOPM

#### 2.2.2. Carboxylation of GO

GO (0.5 g) and 500 mL deionized water were added into a 1000-mL three-neck flask with stirring and dispersing in an ultrasonic water bath for 1.5 h. Then, NaOH (125.0 mmol, 5.0 g) was added into the resulting mixture with stirring by ultrasonic for 30 min. And BrCH_2_COOH (72.0 mmol, 10.0 g) was added into the resulting mixture with stirring. The mixture was stirred at room temperature for 5 h. After the completion of the reaction, the dispersion was centrifuged and washed three times with a mass fraction of 5% diluted hydrochloric acid and distilled water. And the product was dried by a vacuum freeze dryer for 12 h to obtain a carboxylated-modified GO. It was recorded as GO-COOH [12]. The reaction process was shown in.

**Scheme 2.**
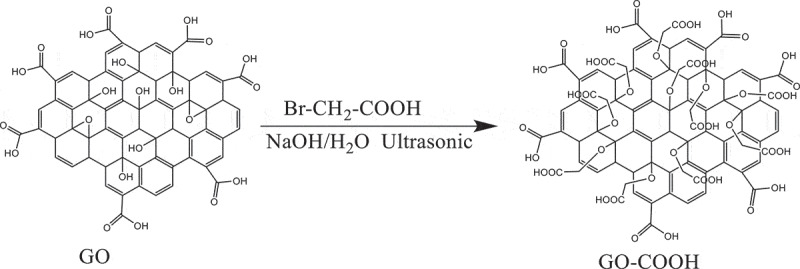
The process of carboxylation modification of GO

#### 2.2.3. Preparation of GO-ODOPM

GO-COOH (1.0 g) was added and dissolved in 300 ml DMF after stirring for 15 min at room temperature. Then, the mixture was stirred at −5°C for 10 min. Then, EDC∙HCL (5.1 mmol, 1.0 g) and DMAP (4.3 mmol, 0.7 g) were successively added into the mixture. After stirring for 30 min, ODOPM (50.0 mmol, 7.0 g) was added into the mixture. The reaction was carried out under the protection of nitrogen atmosphere for 24 h at 30°C. After the completion of the reaction, the product was washed with 5% dilute hydrochloric acid and 5% sodium chloride solution, respectively. And then it was washed with deionized water for three times. The product was freeze-dried for 12 h, and it was recorded as GO-ODOPM. The reaction process was shown in.

**Scheme 3.**
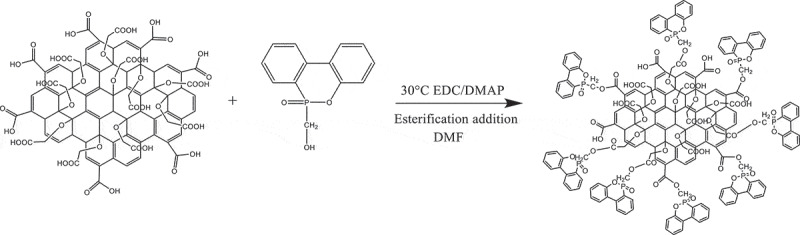
The preparation process of GO-ODOPM

#### 2.2.4. Preparation of GO-ODOPM/PS composite microspheres

One hundred milliliters of deionized water, 10 mL of PVA-124 solution with a mass fraction of 3%, 10 drops of PVA-1788 with a mass fraction of 5% and 30 drops of Na_2_SO_3_ solution with a mass fraction of 3% were added into a 250 ml three-necked flask. And it was heated to 60°Cto dissolve completely under stirring. GO-ODOPM (0 wt%, 0.5 wt%, 1 wt%, 2 wt%, 3 wt%), St (100.0 mmol, 10.4 g), and BPO (20.0 mmol, 5.0 g) were added to the 100-mL beaker and sonicated for 30 min. The oil phase liquid prepared above was added into the three-necked flask and the rotation speed was adjusted to 300 r/min. In the process of polymerization, the strategy of segmented heating was adopted. The reaction was conducted for 1 h at 70°C and 5 h at 80°C. When the microsphere became hard, the temperature was raised to 90°C for 1 h to promote complete monomer transformation. After the completion of the reaction, the temperature of the system was reduced to about 50°C under mechanical agitation, and the liquid in the three-mouth bottle was poured out. Then, the product was washed with hot water for 2–3 times. The product was washed with deionized water and dried with a funnel filter. And then it was dried with an electric blast dryer at 60°C to obtain GO-ODOPM/PS composite microsphere material.

### 2.3. Characterizations

FTIR spectra were obtained with a Spectrum One B FTIR Spectrometer (PE, USA) and recorded between 400 and 4000 cm^−1^. The samples were grinded with KBr and pressed into thin slices. ^1^H-NMR was performed on Bruker AVANCE III-600MHZ HD, DMSO as solvent. X-ray diffraction (XRD) analyses were performed on a PA Nalytical X’Pert PRO diffractometer equipped with Cu-Kα radiation (λ = 0.154 nm). The 2θ range was taken over between 5° and 80° with the scanning rate of 0.02°∙s^−1^. Differential scanning calorimetric (DSC) was carried out in a DSC-204F1 instrument (Netzsch, Germany) at a heating rate of 10°C/min under N_2_ atmosphere (gas flow rate of 40 mL/min). The test temperature range is 25–250°C. TGA was carried out on an STA-4497 thermal analyzer (Netzsch, Germany) under nitrogen. Sample loaded into alumina pan was heated from 25°C to 800°Cat a heating rate of 10°C/min, with a gas flow of 20 mL/min. X-ray photoelectron spectroscopy (XPS) measurement was performed using a Thermo XPS-ESCALAB250Xi electron spectrometer, using AlKα excitation radiation (hν = 1486.6 eV). Raman spectra were recorded by using a labRAMHR-EVdotion Confocal Raman Microprobe using a 532-nm argon-ion laser. Scanning electron microscopy (SEM) images were obtained with a scanning electron microscope S–3400 (Japan) at an accelerating voltage of 10.0 kV. Spraying gold on the surface of the sample. Microscale combustion calorimetry (MCC) was carried out using an FAA Micro calorimeter (FAA Fire testing technology, East Grinstead, UK). The temperature of the pyrolysis zone was from 25°C to 650°C, and the heating rate was 1°C/s. The combustion zone was set at 900°C. Oxygen and nitrogen flow rates were set at 20 and 80 mL/min, respectively.

## 3. Results and discussion

### 3.1. ODOPM structure analysis

**Figure 1.**
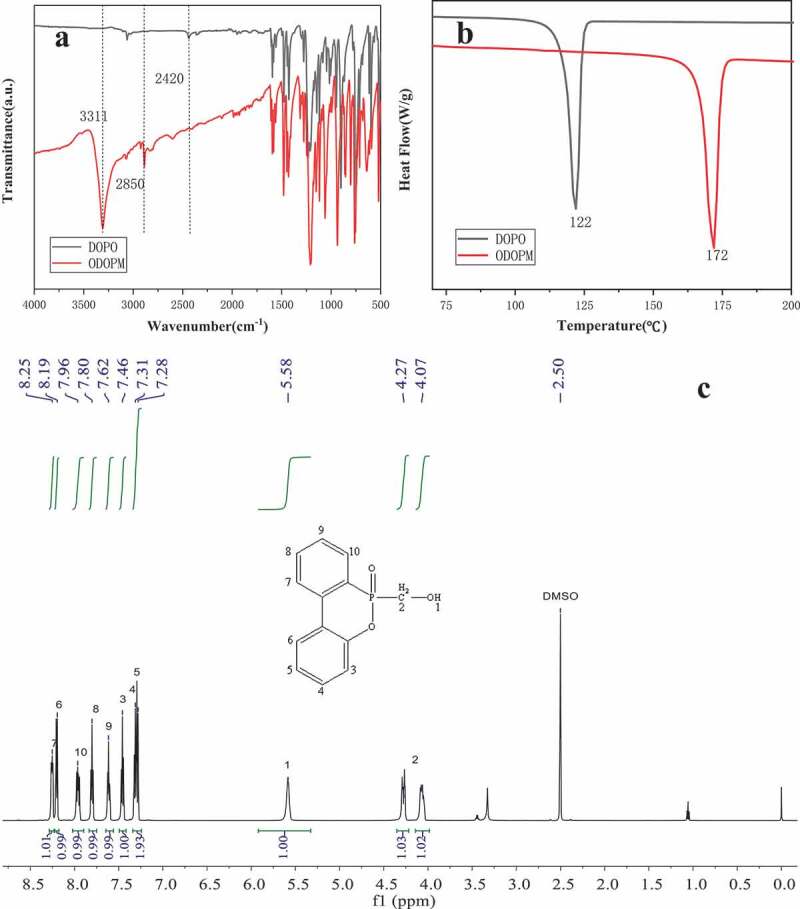
(a) showed the FTIR spectrum of DOPO and ODOPM, (b) showed the DSC spectrum of DOPO and ODOPM, (c) showed the ^1^H-NMR spectrum of ODOPM (600 MHz, DMSO)

The structure of the ODOPM was characterized by FTIR, ^1^H-NMR and DSC. The spectrum is shown in Figure 1. As can be seen in Figure 1(a), the characteristic peaks were displayed at 1209 cm^−1^, 947 cm^−1^, 1429 cm^−1^ and 2420 cm^−1^ were attributed to P = O, P-O-Ph, P-Ph and active P-H bonds in DOPO, respectively. After modified by formaldehyde, the new peaks appeared at 3311 cm^−1^, 2920 cm^−1^, and 2850 cm^−1^ corresponding to the stretching vibration of the hydroxyl group (-OH) and the stretching vibration of the methylene group (-CH_2_), respectively. The special P-H bond at 2420 cm^−1^ disappeared, indicating that DOPO has been successfully hydroxylated [13]. In Figure 1(b), the melting points of the DOPO and ODOPM were compared by DSC. It was shown that the ODOPM had a higher melting point. It was increased by 50°C compared with DOPO, which could further prove that the stable ODOPM was obtained by hydroxylation of DOPO. In Figure 1(c), the structure of the product was further characterized by ^1^H-NMR. Hydrogen in the chemical shift between 7.0 and 8.5 ppm corresponded to eight hydrogens on the benzene ring (ArH, 8H). Hydrogen in the chemical shift between 5.5 and 6.0 ppm corresponded to hydrogen on the hydroxyl group (s, 1H, C-OH), and hydrogen in the chemical shift between 4.0 and 4.5 expected [14].

### 3.2. FTIR analysis

**Figure 2.**
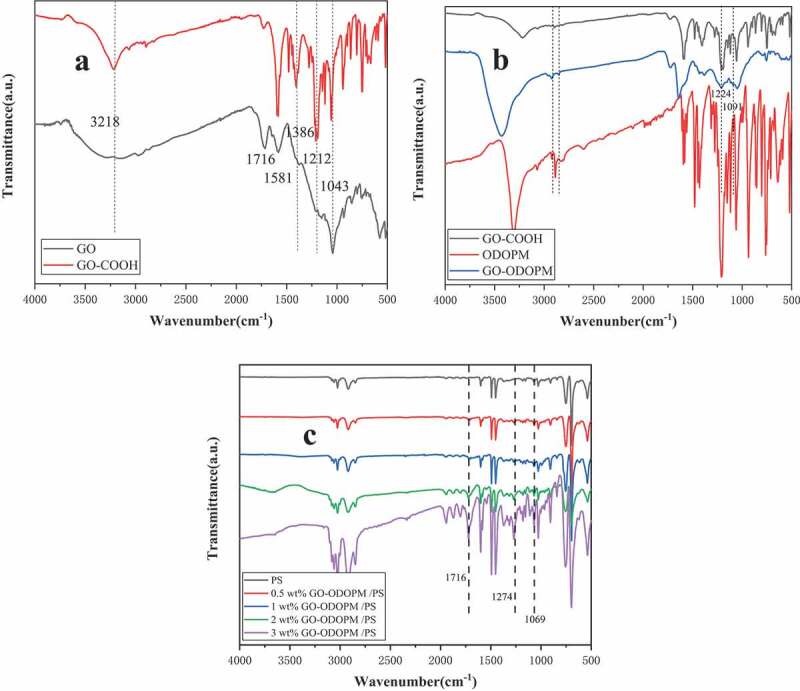
(a) showed the FTIR spectrum of GO and GO-COOH, (b) showed the FTIR spectrum of ODOPM, GO-COOH and GO-ODOPM/PS, (c) showed the FTIR spectrum of GO-ODOPM/PS composite microspheres with different added contents

The GO-COOH, GO-ODOPM and GO-ODOPM/PS composite microspheres were characterized by FTIR, and the spectrums are shown in Figure 2. In Figure 2(a), a large and broad peak appeared at 3000–3500 cm^−1^. This peak corresponded to the stretching vibration of the -OH bond in the carboxyl group and the hydroxyl group on GO. The peaks at 1716 and 1581 cm^−1^ corresponded to the stretching vibration of C = O bond in the carboxyl group, and the peaks near 1043 cm^−1^ corresponded to the stretching vibration of C-O-C in the epoxy group. However, after carboxylation, the broad peak became a sharp peak near 3218 cm^−1^, which corresponded to the stretching vibration of the -OH bond on the carboxyl group. In addition, the stretching vibration of methylene (-CH_2_) occurred at 2850 and 2920 cm^−1^. The bending vibration of methylene (-CH_2_) occurred at 1396 cm^−1^, and the new ether bond occurred at 1212 cm^−1^ (-C-O-C-) and the decrease of the absorption peak intensity of epoxy group at 1043 cm^−1^ fully demonstrated that GO was successfully modified to obtain GO-COOH [15]. In Figure 2(b), compared with GO-COOH, the stretching vibration peak of the -CH_2_ corresponded to GO-ODOPM (at 2850 and 2920 cm^−1^) and the stretching vibration peak of the C = O bond in the ester group corresponded to 1716 and 1581 cm^−1^. The stretching vibration peak was significantly enhanced. In addition, P = O and P-O-Ph bonds in ODOPM were found at 1274 and 1091 cm^−1^, indicating that ODOPM was grafted to GO-COOH through covalent bonds [16]. In Figure 2(c), the infrared spectra of GO-ODOPM/PS composite microspheres with different addition amounts were characterized. It can be seen from the Figure that compared with the pure PS microspheres, as the amount of GO-ODOPM increased, the stretching vibration of C = O bond, P = O bond and P-O-Ph bond in the corresponding ester groups of GO-ODOPM/PS microspheres at 1716, 1274 and 1069 cm^−1^ also became stronger.

### 3.3. XPS analysis

**Figure 3.**
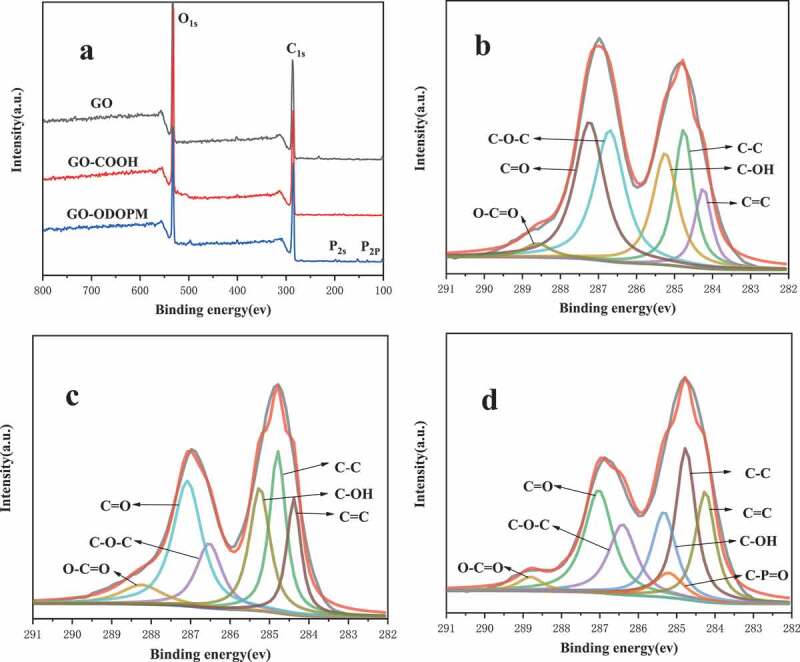
The XPS (a) survey spectra of GO, GO-COOH and GO-ODOPM. High-resolution XPS spectra of (b) C1s for GO, (c) C1s for GO-COOH, (d) C1s for GO-ODOPM

**Figure 4.**
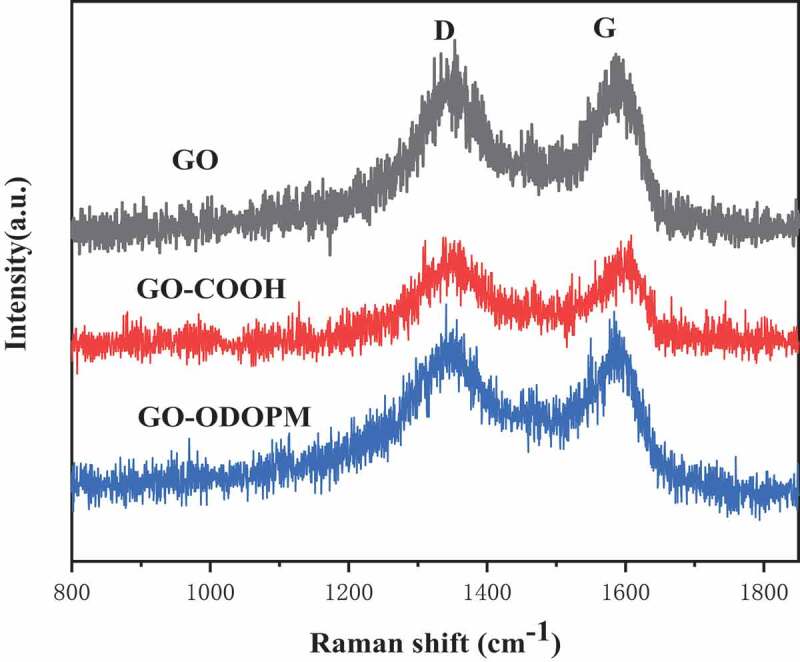
Raman spectrum of GO, GO-COOH and GO-ODOPM

In order to better study the changes of GO, GO-COOH and GO-ODOPM surface atoms and functional groups. In this paper, full-spectrum scanning and C1_S_ scanning were performed on GO, GO-COOH and GO-ODOPM by XPS. The spectrum is shown in Figure 3. It can be seen in Figure 3(a) that GO and GO-COOH only showed two characteristic peaks of C1s and O1s. However, GO-ODOPM showed two new peaks at 134.6 and 190.4 eV, respectively, corresponding to P2p and P2s characteristic peaks in ODOPM. The contents of GO, GO-COOH and GO-ODOPM surface elements are given in Table 1. It can be seen that the content of oxygen elements was slightly increased after carboxylation modification, which was mainly caused by the conversion of hydroxyl and epoxy groups on GO into carboxyl groups. In the GO-ODOPM, the phosphorus content changed from 0 to 2.42, indicating that ODOPM was successfully grafted onto the GO-COOH. Figure 3(b,Figure 3c) shows the C1s high-resolution spectra of GO and GO-COOH, respectively. The deconvoluted C1s XPS spectrum of GO and GO-COOH was associated with Six bands: C-C (284.8 eV), C = C (284.2 eV), C-OH (285.2 eV), C-O-C (286.7 eV), C = O (287.2 eV), O-C = O (288.4 eV), which was consistent with other studies presented in the literature [17]. It can be seen that the type of peak of GO-COOH by carboxylation modification was the same as that of GO, but it was worth noting that the integral area of the two peaks, C-OH (285.2 eV) and C-O-C (286.7 eV) decreased significantly in GO-COOH, corresponding to the reduction of surface hydroxyl and epoxy groups, which also corresponded to the FTIR results in the Figure. Figure 4(d) shows the C1s high-resolution spectra of GO-ODOPM. The deconvoluted C1s XPS spectrum of GO-ODOPM was associated with Seven bands: C-C (284.7 eV), C = C (284.3 eV), C-OH (285.3 eV), C-O-C (286.4 eV), C = O (287 eV), O-C = O (288.9 eV), C-P = O (285.2 eV). But it should be noted that the characteristic peak of C-P = O bond in ODOPM was observed at 285.2 eV. In addition, O-C = O (288.9 eV) in GO-ODOPM moved to a higher position than GO-COOH, which also proved that ODOPM was covalently grafted on the surface of GO [10].

**Table 1.** Contents of various elements on the surface of GO, GO-COOH and GO-ODOPM

Sample	O (%)	C (%)	P (%)
GO	30.18	69.82	0
GO-COOH	32.44	67.56	0
GO-ODOPM	29.36	68.22	2.42

### 3.4. Raman analysis

Figure 4 shows the Raman spectrum of GO, GO-COOH, GO-ODOPM, and the data were shown in Table 2. It can be seen that all three materials exhibited two prominent peaks at 1340 and 1586 cm^−1^, which correspond to the D and G peaks of the graphene material, respectively. The D and G bands are assigned to the breathing mode of k-point mode of A 1 g symmetry and the E 2 g vibration mode of sp^2^ hybridized carbons in the graphite crystal domain, respectively, [18]. In the Raman spectrum of graphene materials, we generally use the ratio of the integrated intensities/area of D to G band (I_D_/I_G_) to characterize the defects of the material. Generally, we think that the larger the ratio of I_D_/I_G_, the more the group was introduced on the surface, and the more serious the defects appeared in the corresponding materials [19]. In Table 2, we can see that GO has many hydroxyl, carboxyl and epoxy groups on its surface, and the integrated area ratio of I_D_/I_G_ was 1.02. However, for GO-COOH, the hydroxyl and epoxy groups on the surface of GO were converted into carboxyl groups. The reduction of the number of hydroxyl and epoxy groups leads to the reduction of surface defects, as a result, the integral area ratio of I_D_/I_G_ was reduced to 0.79. When ODOPM was grafted onto GO-COOH, its defects increased due to its large benzene ring, so that the integral area ratio of I_D_/I_G_ increased to 1.86. These results also proved that the ODOPM group was grafted to the GO-COOH surface, which was consistent with the results of FTIR and XPS characterization.

**Table 2.** Data of Raman spectra of GO, GO-COOH and GO-ODOPM

			Integral area		
Sample	X_D_ (cm^−1^)	X_G_ (cm^−1^)	D	G	I_D_/I_G_
GO	1340	1586	6102	5990	1.02
GO-COOH	1350	1603	7576	9515	0.79
GO-ODOPM	1338	1590	8679	4654	1.86

### 3.5. Morphological analysis

**Figure 5.**
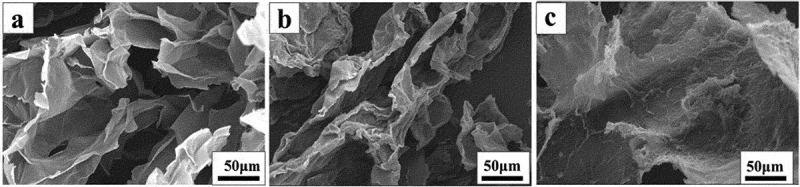
SEM image of GO, GO-COOH and GO-ODOPM

The surface morphology of GO, GO-COOH and GO-ODOPM was analyzed by SEM. In Figure 5(a), we can see that GO showed a layered structure with many thin layers stacked together, and the surface of the slices was relatively smooth. In Figure 5(b), GO-COOH still presented a layered structure. The difference is that the thickness of GO-COOH sheet increased slightly, which was mainly due to the replacement of hydroxyl and epoxy groups on GO sheet by -O-CH_2_-COOH groups, resulting in the increase of thickness of GO-COOH sheet [20]. In Figure 5(c), it can be seen that the thickness of GO-ODOPM sheet is obviously increased, and the sheet was rough and curly. This is mainly due to the large steric hindrance of ODOPM, which occupied a larger space volume and inhibited the stacking of slices. This further proved that the ODOPM group was grafted to the GO-COOH surface.

### 3.6. XRD analysis

**Figure 6.**
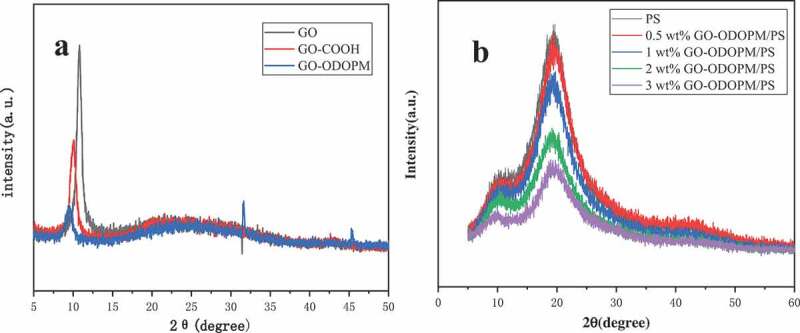
(a) showed the XRD spectrum of GO, GO-COOH and GO-ODOPM, (b) is the XRD spectrum of GO-ODOPM/PS composite microspheres with different addition amounts

In order to further verify the structure of GO-COOH, GO-ODOPM and the dispersion state of GO-ODOPM in GO-ODOPM/PS composite microspheres. XRD analysis was performed on GO-COOH, GO-ODOPM and different content of GO-ODOPM/PS composite microspheres. The spectrum is shown in Figure 6. In Figure 6(a), we can see that GO presented a sharp characteristic peak at 2θ = 10.84°, corresponding to the (002) diffraction peak in the graphite lattice. After carboxylation, the characteristic peak of GO-COOH moved to 9.96 and the peak intensity decreased. This is because the hydroxyl group and epoxy group on GO were converted into carboxyl groups, which leads to an increase in the distance between the graphene sheets. From the Bragg equation λ=2dsinθ, it can be seen that the diffraction angle moves to the low angle direction when the interlayer distance increases. When the ODOPM was grafted, the distance between the layers of GO-ODOPM further increased, the 002 characteristic peak of GO shifted to 9.4° and the peak intensity greatly decreased, which inhibited the accumulation between the layers of graphene [21]. Figure 6(b) shows the XRD spectra of different additions of GO-ODOPM/PS composite microspheres. It can be seen that the two broad characteristic peaks of pure PS microspheres at 2θ = 10.3° and 19.8° correspond to the amorphous characteristic diffraction peaks of PS, respectively. After adding GO-ODOPM, the characteristic peak of GO-ODOPM was not found in the XRD spectrum. The characteristic peak position of polystyrene did not change, but the intensity of the peak decreased, indicating that GO-ODOPM was well dispersed in the PS matrix [22].

### 3.7. DSC analysis

**Figure 7.**
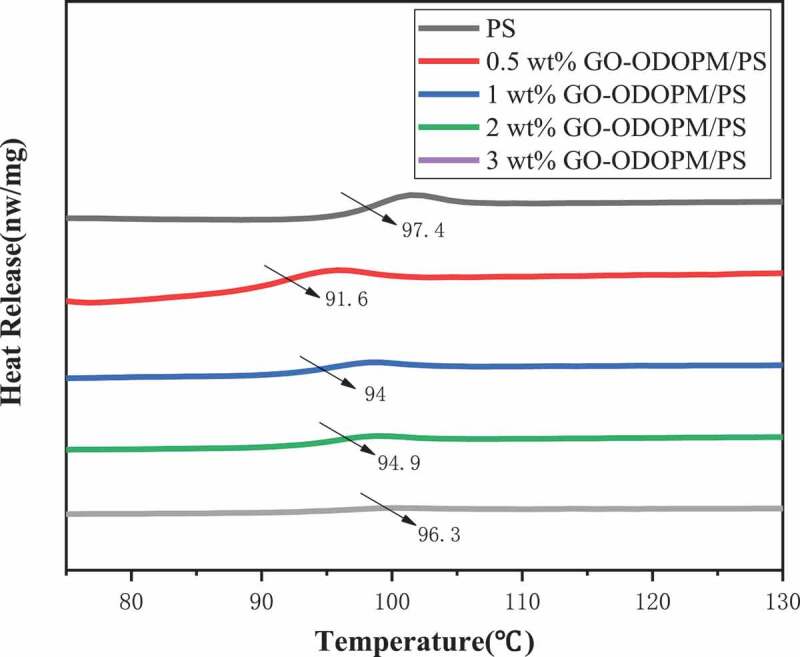
T_g_ of GO-ODOPM/PS composite microspheres in different amounts

The glass transition temperature (T_g_) of GO-ODOPM/PS composite microspheres with different content of GO-ODOPM was studied by DSC. The spectrum is shown in Figure 7. In Figure 7, we can see that the T_g_ of pure PS microspheres was 97.4°C. After adding GO-ODOPM, the T_g_ of the composites decreased overall, but interestingly, as the amount of GO-ODOPM added increased, T_g_ showed an upward trend. This may be due to the fact that when the addition amount of GO-ODOPM was low, the ODOPM group acts as a lubricant in the composite material, which increased the flexibility of the polystyrene chain, resulting in a decrease in the T_g_ of the GO-ODOPM/PS composite microsphere [23]. With the increase of the addition amount of GO-ODOPM, GO sheets played a major role in the composite microspheres, which can inhibit the movement of PS molecular segments through interface interactions such as physical adsorption, and act as plasticizers, resulting in the increase of T_g_ of GO-ODOPM/PS composite microspheres [24].

### 3.8. Thermogravimetric analysis (TGA)

**Figure 8.**
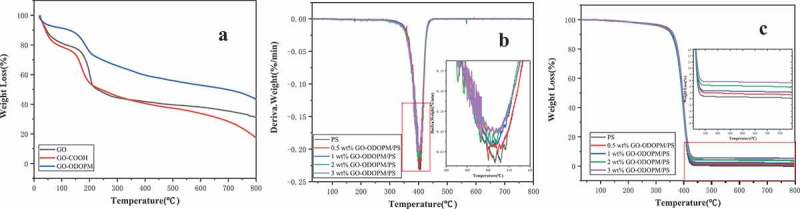
(a) showed the TG spectra of GO, GO-COOH and GO-ODOPM, (b) and (c) were, respectively, the TG and DTG spectra of GO-ODOPM/PS microspheres with different addition amounts

The thermal stability of GO, GO-COOH, GO-ODOPM and GO-ODOPM/PS composite microspheres was studied by TGA. The spectra under the N_2_ atmosphere are shown in Figure 8. It can be seen from Figure 8(a) that the mass loss of these three substances can be roughly divided into three stages. The mass loss in the first stage mainly occurred below 100°C, mainly due to the evaporation of physically adsorbed water in the sample. This is mainly because there are many hydrophilic groups on GO, which have strong hygroscopicity and can absorb moisture in the air. The second stage of mass loss mainly occurred between 120°C and 200°C, which mainly corresponds to the decomposition of oxygen-containing functional groups. Compared with GO, GO-COOH showed a higher mass-loss rate, indicating that GO-COOH contained more -COOH groups. However, GO-ODOPM showed a low-mass loss rate, indicating that the carboxyl group on the surface of GO-COOH was greatly reduced by the carboxyl group reaction between ODOPM and GO-COOH, which greatly improved its thermal stability. The third stage occurs after 200°C, which can be attributed to the high-temperature carbonization decomposition of the carbon layer on GO [25]. When the temperature reaches 800°C, GO-ODOPM still retains a higher mass residual ratio than GO and GO-COOH, indicating that its thermal stability is greatly improved. Figure 8(b,Figure 8c) showed the TG and DTG of different content of GO-ODOPM/PS microspheres. In Figure 8(b,Figure 8c), we can see that both pure PS microspheres and different content of GO-ODOPM/PS microspheres presented the first-order decomposition. The initial decomposition temperature of GO-ODOPM/PS composite microspheres took precedence over pure PS microspheres, but with the increase of GO-ODOPM content, the maximum weight loss rate gradually decreased, and the remaining residual carbon content gradually increased. This is mainly due to the fact that during thermal oxidative degradation, ODOPM has lower thermal stability and preferential decomposition, which can promote the coking of PS carbon chain into carbon. In addition, good dispersion and interfacial interaction between the GO sheet and the PS matrix, as well as the large specific surface area of the GO sheet enhanced heat transfer. The GO sheet acted as a physical barrier, providing sufficient time to capture degraded polymer radicals to inhibit thermal oxidative degradation and improving the thermal stability of the PS composite [26].

### 3.9. Micro-scale combustion calorimetry analysis (MCC)

**Figure 9.**
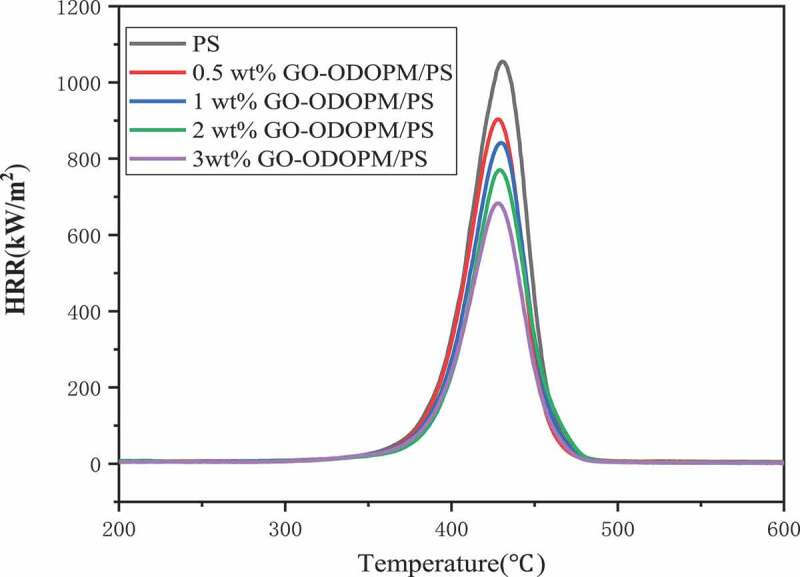
Heat release rate of GO-ODOPM/PS composite microspheres with different additions

In order to further study the combustion properties of PS microspheres and their composites, the MCC was used to characterize the combustion properties [27]. The curves of the heat release rate (HRR) versus temperature for PS and different additions of GO-ODOPM/PS composite microspheres are given in Figure 9, and the corresponding data are listed in Table 3. We can see that the pure PS microspheres burnt rapidly, and the PHRR and THR values were 1022.7 W/g and 47 kJ/g, respectively. With the increase of the addition amount of GO-ODOPM from 0.5 wt% to 3 wt%, the PHRR of GO-ODOPM/PS composite microspheres gradually decreased by 14.3%, 20.7%, 28.4% and 36.2%, respectively. The THR value also showed a similar trend to PHRR, and the addition of 3 wt% GO-ODOPM resulted in the lowest THR (33.6% reduction). The reduced THR value indicated less heat release during combustion, which was advantageous for inhibiting combustion. For heat release capacity (HRC), as a good predictor of fire and flammability behavior, it was obtained by dividing the peak heat release rate by the heating rate and represented the maximum capacity at which the material releases the heat of combustion. We can see that in pure PS microspheres, the HRC can be as high as 1000 J/g·k^−1^. With the increase of GO-ODOPM, the HRC value decreased from 1000 J/g·k^−1^ (PS) to 645 J/g·k^−1^ (3 wt% GO-ODOPM/PS), which can be attributed to the decrease of the maximum mass loss rate and the reduction of combustion heat of decomposition products at this temperature [28]. In the GO-ODOPM/PS composite microsphere system, the ODOPM decomposed during combustion to produce phosphorus-containing radicals and polyphosphoric acid. These high-concentration free radicals destroyed the adhesion of the substrate surface and eliminated hydrogen and oxygen-free radicals. And they realized radical quenching. They catalyzed carbon formation and inhibited flame combustion to protect the substrate. On the other hand, good dispersion and interfacial interaction between the GO sheet and the PS matrix, as well as the large specific surface area of the GO sheet enhanced heat transfer. The GO sheet acted as a physical barrier, providing sufficient time to capture degraded polymer radicals to inhibit thermal oxidative degradation. This was consistent with the improvement of the carbon residue rate in TG. It achieved the synergistic flame retardancy between phosphorous flame-retardancy agent and inorganic nano flame-retardancy agent and played a good role in flame-retardancy [26].

**Table 3.** GO-ODOPM/PS microsphere MCC test data with different addition amounts

Samples	PHRR(W/g)	THR(KJ/g)	HRC(J/g·k^−1^)	T_max_ (°C)
PS	1022.7	47	1000	427.9
0.5 wt% GO-ODOPM/PS	876.2	39.9	857	427
1 wt% GO-ODOPM/PS	811.1	37.5	790	426.5
2 wt% GO-ODOPM/PS	732.5	34.2	720	426.1
3 wt% GO-ODOPM/PS	652.6	31.2	645	425.7

### 3.10. Char residues analysis

**Figure 10.**
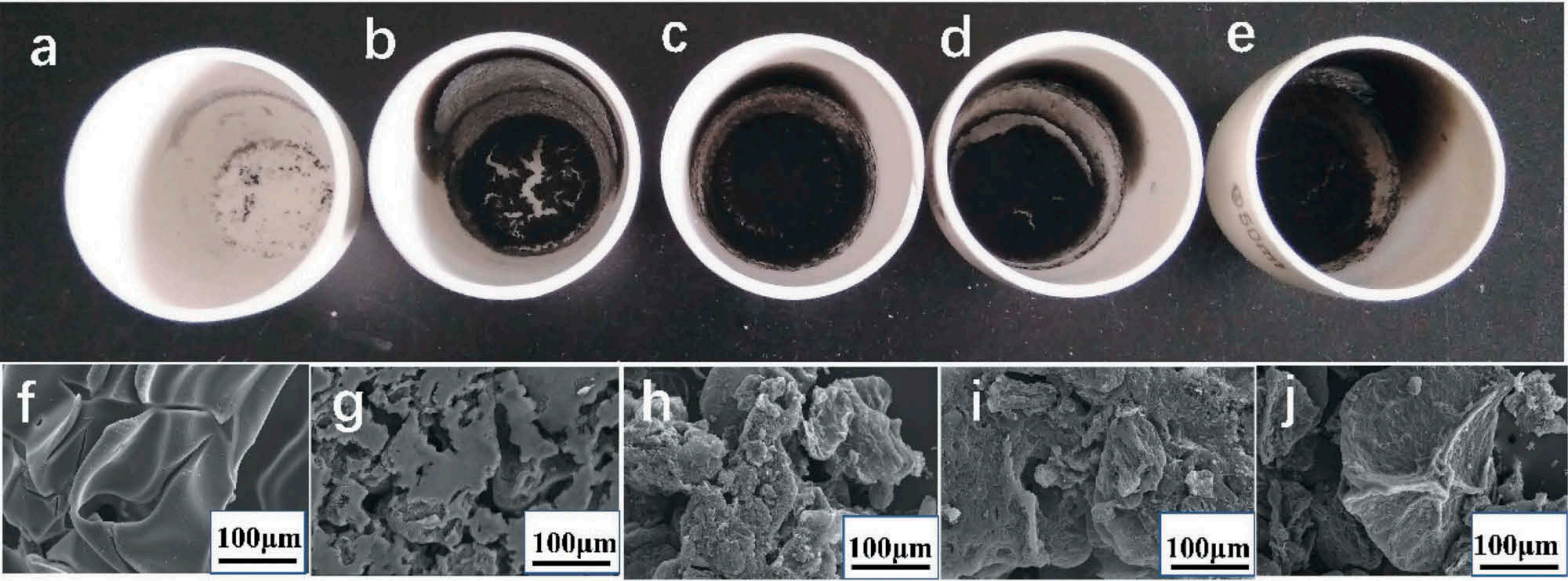
Digital photos of the char residues of (a) neat PS microspheres, (b) 0.5 wt% GO-ODOPM/PS, (c) 1 wt% GO-ODOPM/PS, (d) 2 wt% GO-ODOPM/PS and (e) 3 wt% GO-ODOPM/PS. SEM images of the char residues from, (f) neat PS microspheres, (g) 0.5 wt% GO-ODOPM/PS, (h) 1 wt% GO-ODOPM/PS, (i) 2 wt% GO-ODOPM/PS and (j) 3 wt% GO-ODOPM/PS

Figure 10(a–Figure 10e) presented the digital photos of the residual char of PS microspheres and different additions of GO-ODOPM/PS composite microspheres after the Combustion test. For pure PS microspheres, the char residues were very weak, while with the increase of the amount of GO-ODOPM, the amount of carbon residue after combustion also increased gradually. Particularly, 3 wt% GO-ODOPM/PS composite microspheres made a more compact and continuous char surface and the amount of char residues was remarkably increased. SEM images allow us to observe microstructures of external char residues on a microscopic scale, in order to further elucidate the effect of GO-ODOPM on the char-forming capability of PS composite microspheres. As shown in Figure 10(f–Figure 10j), for pure PS microspheres, the residual carbon surface was relatively smooth and there were many large and deep cracks. With the increase of the amount of GO-ODOPM, the surface of the carbon layer was covered by phosphoric-oxygenic compound, which became rough and dense. Particularly, the char residues of 3 wt% GO-ODOPM/PS composite microspheres exhibited a more cohesive and compressed layer on the surface without obvious holes and cracks, indicating relatively slow transfer of heat and combustible gases during thermal degradation and combustion by introducing 3 wt% GO-ODOPM, and it is beneficial to suppress the combustion process [29].

**Figure 11.**
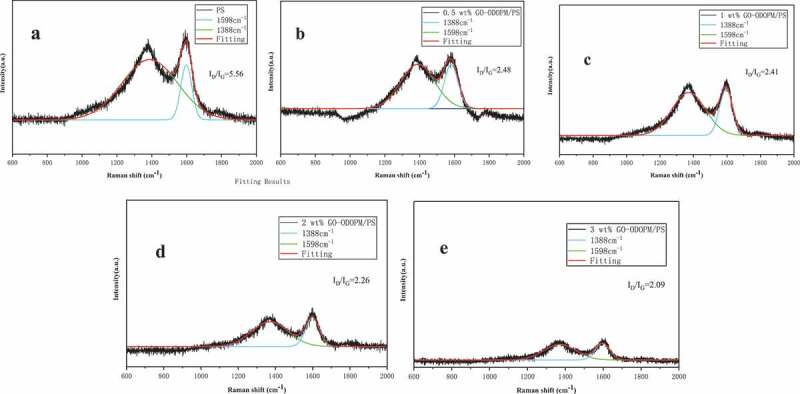
Raman spectra of the char residues of (a) neat PS microspheres, (b) 0.5 wt% GO-ODOPM/PS, (c) 1 wt% GO-ODOPM/PS, (d) 2 wt% GO-ODOPM/PS and (e) 3 wt% GO-ODOPM/PS

Figure 11(a–Figure 11e) presented the Raman spectra of the char residues for PS microspheres and different additions of GO-ODOPM/PS composite microspheres after the Combustion test and the data was shown in Table 4. All the spectra showed two broad peaks at 1375 cm^−1^ and 1598 cm^−1^, corresponding to the D and G bands, respectively. The D and G bands were assigned to the breathing mode of k-point mode of A 1 g symmetry and the E 2 g vibration mode of sp_2_ hybridized carbons in the graphite crystal domain, respectively. The ratio of the integrated intensities of D to G band (I_D_/I_G_) was used to assess the graphitization degree of the char residues [30]. It can be obviously seen that the I_D_/I_G_ ratio significantly decreased from 5.56 (pure PS microspheres) to 2.09 (3 wt% GO-ODOPM/PS composite microspheres), indicating that the char residues of GO-ODOPM/PS composite microspheres showed a higher graphitization degree than pure PS composite microspheres [31]. The high graphitization degree of 3 wt% GO-ODOPM composite microspheres can be attributed to the synergistic effect between the catalytic action of ODOPM and the physical effect of graphene sheets, towards forming more stable char structures.

**Table 4.** GO-ODOPM/PS composite microsphere Raman spectra data with different addition amounts

			Integral intensity		
Sample	X_D_ (cm^−1^)	X_G_ (cm^−1^)	D	G	I_D_/I_G_
PS	1388	1598	84.75	15.24	5.56
0.5 wt% GO-ODOPM/PS	1389	1581	71.26	28.73	2.48
1 wt% GO-ODOPM/PS	1371	1594	70.65	29.34	2.41
2 wt% GO-ODOPM/PS	1370	1598	69.39	30.60	2.26
3 wt% GO-ODOPM/PS	1371	1595	67.72	32.27	2.09

### 3.11. Rheological behavior analysis

**Figure 12.**
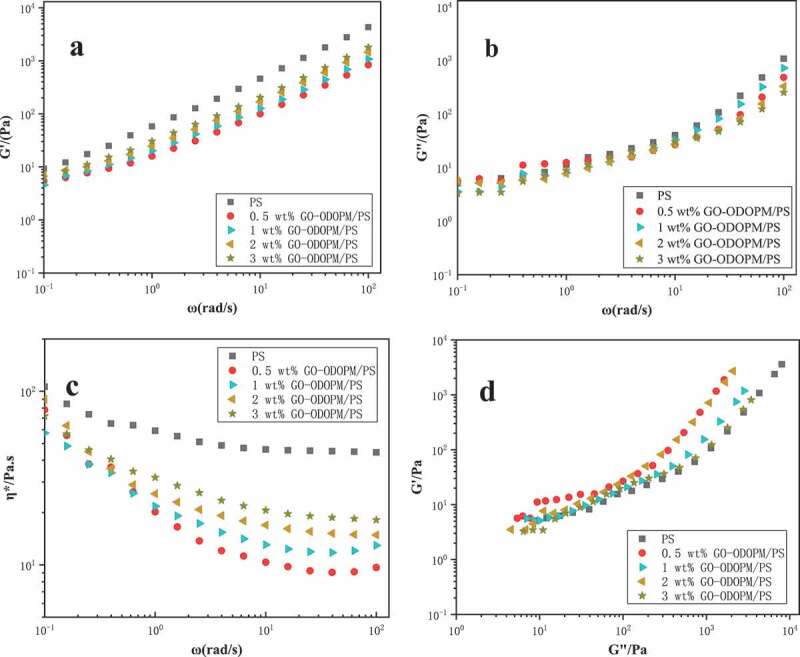
(a), (b) and (c), respectively, show the relation curves of energy storage modulus, loss modulus, complex viscosity and frequency (ω) of GO-ODOPM/PS microspheres with different addition amounts, (d) shows the double logarithm curves of G′ and G″ at 190°C of GO-ODOPM/PS microspheres with different addition amounts

Polymer rheology is an important method to study the structure and properties of polymers and their nanocomposites. Through dynamic rheological analysis, information on viscoelasticity and structural changes of polymers during processing can be obtained. In recent years, rheological studies have been used to study the flame-retardant mechanism and carbon formation mechanism of nano-flame retardant composite materials. And it has become a research hotspot in the field of flame-retardant [32]. In this paper, dynamic frequency scanning was performed on pure PS microspheres and different content of GO-ODOPM/PS microspheres at 190°C using a rotational rheometer. In Figure 10, the curves of storage modulus (G′), loss modulus (G″), complex viscosity (η) and frequency (ω) of PS microspheres and GO-ODOPM/PS composite microspheres with different content and the double logarithm curves of G′ and G″ at 190°C are given. In Figure 12(a), it can be seen that in the test frequency range (0.1–200 rad/s), the G’ of the flame-retardant system was lower than that of the pure PS microspheres, and it gradually increased with increasing of the flame-retardant content. At low frequencies, the appearance of the energy storage modulus platform indicated the change of GO-ODOPM/PS composite microsphere structure and the formation of a three-dimensional network structure [33]. This was mainly because ODOPM has a large spatial effect and can reduce the entanglement between the PS chain segments. When the content of GO-ODOPM was low, the ODOPM group played a leading role in it, which weakened the entanglement between the PS molecular chains and increased the kinetic ability of the molecular chains, acting as a lubricant. When the content of GO-ODOPM increased, the nano-layered GO sheet played a leading role. When the content of GO-ODOPM increased, GO sheets with nano-layered structure played a leading role in it, and the molecular chain would be restricted by GO sheets to play the role of physical entanglement, leading to the enhanced elasticity of GO-ODOPM/PS composite microspheres and the increase of G’ value. This was also consistent with the results of the DSC characterization described above. In Figure 12(b), it can be seen that the G′′ of the flame-retardant system was lower than that of the pure PS microspheres, indicating that the energy loss of GO-ODOPM/PS composite microspheres during viscous deformation was lower than that of pure PS microspheres, which was beneficial to resist the influence of external thermal environment. In Figure 12(c), the complex viscosity (η) of GO-ODOPM/PS composite microspheres was lower than that of PS microspheres. With the increasing amount of GO-ODOPM, the complex viscosity showed an increasing trend. This was mainly because that the GO sheets which were uniformly dispersed in the PS matrix constituted an entanglement point hindered the movement of the PS segment and caused the increase of the melt viscosity. When the shearing frequency increased, the network structure formed was destroyed. When the time of the shear stress was less than the relaxation time of the macromolecular chain, the macromolecular chain did not have enough time to shrink, resulting in the melt flow resistance and the viscosity decreased rapidly, which showed the behavior of shear-thinning [34]. Figure 12(d) shows the double logarithm relation curve of G’ and G” of GO-ODOPM/PS microspheres with different addition amounts. It is also known as the Han relation curve, which was used to qualitatively determine the compatibility and viscoelastic properties of melt blends. In the Figure, the curve of the GO-ODOPM/PS composite microsphere and the curve of the pure PS microsphere were generally consistent, indicating that the system had a good compatibility in the mixing process and no phase separation [35]. Generally, when the Han curve is located on the right side of the diagonal line, the viscosity is dominant, or the elasticity is correspondingly dominant. After GO-ODOPM was added, the system changed from the dominant viscous response to the dominant elastic response, and as the amount of GO-ODOPM increased, it changed to the dominant viscous response. It can be seen from the above rheological test data that the addition of GO-ODOPM could increase the storage modulus and complex viscosity of GO-ODOPM/PS composite microspheres and reduce their loss modulus. This indicated that the internal structure of GO-ODOPM/PS composite microspheres has changed with the increasing amount of GO-ODOPM. And the polymer has changed from liquid-like to solid-like, forming a three-dimensional network inside the polymer. This also indicated that the addition of GO-ODOPM could improve the thermal and flame-retardant properties of GO-ODOPM/PS composite microspheres and improve the resistance of the PS chain segment to thermal degradation. It also protected the inner carbon layer, which further verified the above DSC and TG test results.

### 3.12. Flame-retardant mechanism analysis

**Figure 13.**
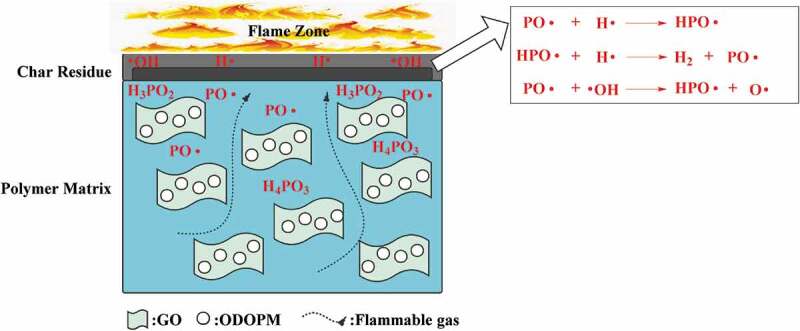
Flame-retardant mechanism of GO-ODOPM/PS composite microspheres

Through the analysis of TG, MCC, Dynamic rheological analysis and char residues of GO-ODOPM/PS microspheres with different contents, it can be seen that the GO-ODOPM could significantly improve the flame-retardant performance of PS composite materials. In Figure 13, the possible flame-retardant mechanism of GO-ODOPM/PS composite microspheres was proposed. GO-ODOPM mainly achieved flame-retardant effect by gas-phase free radical quenching and condensed phase catalyzed carbonization [36]. Gas-phase flame-retardant mechanism can be explained as that free radical chain reactions such as chain initiation, chain growth, chain transfer and chain termination in the process of polymer thermal degradation, which could effectively achieve flame retardant by interfering with the free radical chain reaction of polymer. In the process of polymer combustion, the gaseous products formed by thermal decomposition of ODOPM contained PO∙ and HPO∙. These low-energy free radicals could capture H∙ and HO∙ radicals in the process of chain growth and chain transfer reaction, thus inhibiting chain growth reaction in the combustion process, reducing the number of free radicals needed to maintain the combustion reaction and preventing the combustion of the polymer [37]. The mechanism of condensed phase catalytic carbonization can be explained as that the GO sheets in GO-ODOPM could reduce the permeability of combustible gas in GO-ODOPM/PS composite microspheres during thermal degradation. The ODOPM groups can generate phosphorus-containing substances such as H_3_PO_2_, H_4_PO_3_ and the like during pyrolysis, thus achieving the effect of catalyzing carbon residues to form carbon, forming a continuous compact carbon layer, and effectively blocking heat transfer and combustible gas transmission [38]. Therefore, the GO-ODOPM/PS composite microspheres have better flame-retardant performance, and the flame-retardant performance was better with the increase of the amount of GO-ODOPM added.

## 4. Conclusions

In this work, a phosphorus nano-flame retardant (GO-ODOPM) through the functional modification of GO with DOPO was successfully fabricated, and then the GO-ODOPM was incorporated into PS microspheres system to prepare fire-resistant GO-ODOPM/PS composite microspheres. The flame retardancy of PS composite microspheres was enhanced by the addition of GO-ODOPM. The results showed that the thermal stability of GO-ODOPM/PS composite increased after adding GO-ODOPM, and the carbon residue gradually increased with the increase in the addition amount. The decrease of the peak heat release rate (HRR) and total heat release rate (THR) for the GO-ODOPM/PS composite microspheres was obtained when the content of the additives was only 3.0 wt% is more than 36.2% and 33.6% compared with the pure PS microspheres, respectively. Through the analysis of the flame-retardant mechanism of GO-ODOPM/PS composite microspheres, it can be seen that the flame-retardancy mechanism of GO-ODOPM in PS microspheres was based on the synergistic effect between the catalytic action of ODOPM and the physical effect of graphene sheets. Thus, this work paves a feasible pathway to design efficient flame retardants for enhancing fire safety of polymers.
